# A Study of *JUN*’s Promoter Region and Its Regulators in Chickens

**DOI:** 10.3390/genes15101351

**Published:** 2024-10-21

**Authors:** Ruihong Kong, Jieyao Shi, Ke Xie, Han Wu, Xu Wang, Yani Zhang, Yingjie Wang

**Affiliations:** 1 Jiangsu Key Laboratory of Sericultural and Animal Biotechnology, School of Biotechnology, Jiangsu University of Science and Technology, Zhenjiang 212100, China; 221211802109@stu.just.edu.cn (R.K.); 222211801210@stu.just.edu.cn (J.S.); 212211801232@stu.just.edu.cn (K.X.); 231211802114@stu.just.edu.cn (H.W.); 212211801132@stu.just.edu.cn (X.W.); 2 Key Laboratory of Silkworm and Mulberry Genetic Improvement, Ministry of Agriculture and Rural Affairs, Sericultural Scientific Research Center, Chinese Academy of Agricultural Sciences, Zhenjiang 212100, China; 3 College of Animal Science and Technology, Yangzhou University, Jiangsu Province Key Laboratory of Animal Breeding and Molecular Design, Yangzhou 225009, China; ynzhang@yzu.edu.cn

**Keywords:** *JUN*, promoter, regulation, JNK pathway, transcription factor

## Abstract

**Background:** The Jun proto-oncogene (*JUN*), also referred to as *C-JUN*, is an integral component of the JNK signaling pathway, which is crucial for the formation and differentiation of spermatogonial stem cells (SSCs). Investigations into the transcriptional regulation of chicken *JUN* can offer a molecular foundation for elucidating its mechanistic role in SSCs. **Methods:** In this study, we successfully cloned a 2000 bp upstream sequence of the *JUN* transcription start site and constructed a series of pGL3 recombinant vectors containing *JUN* promoters of varying lengths. **Results:** We verified the promoter activity of the 2000 bp upstream sequence by assessing the fluorescence intensity of DF-1 and identified the promoter activities of different regions via dual-luciferase assays. The transcription of JUN and its promoter region spanning −700 to 0 bp was modulated by an activator of the JNK signaling pathway. Bioinformatics analysis revealed that this −700 to 0 bp region was highly conserved among avian species and predicted the presence of binding sites for Wilms tumor 1 (*WT1*) and CCAAT/enhancer binding protein alpha (*CEBPA*). The JNK signaling pathway activator was found to upregulate the expression of these transcription factors in DF-1 cells. Through the deletion of binding sites and the overexpression of *WT1* and *CEBPA*, we demonstrated that *WT1* inhibited the transcription of *JUN*, while *CEBPA* promoted it. **Conclusions:** In conclusion, the −700 to 0 bp region is the key region of the *JUN* promoter, with *WT1* inhibiting *JUN* transcription. The results of the study not only provide ideas for exploring the regulatory mechanism of *JUN* in chicken SSCs, but also lay an important foundation for the study of avian SSCs.

## 1. Introduction

The Jun proto-oncogene (*JUN*), also referred to as *C-JUN*, is a critical component of the *AP-1* transcription factor complex and serves as the most potent transcriptional activator within the *JUN* family. It modulates the expression of target genes by binding to their promoters or enhancers, thereby playing a pivotal role in cellular processes such as proliferation [1], differentiation [2], and apoptosis [3]. Evidence suggests that *JUN* exhibits differential expression across various types of mice spermatogonia [4] and is involved in regulating germ cell apoptosis through Sertoli cells [5]. Additionally, JUN has been observed to colocalize with the essential germ cell factor Dead end 1 β (DND1-β) within the nuclei of GC-1 spermatogonia cells [6]. The down-regulation of JUN could inhibit the apoptosis of Spermatogonial stem cells (SSCs) [7]. A differential expression of *JUN* was observed between chicken embryonic stem cells (ESCs) and SSCs [8]. Furthermore, *JUN* plays a regulatory role in the transcription of gga-miR-31-5p, which has been implicated in the differentiation of chicken SSCs [9].

Current research predominantly focuses on humans and mice to elucidate the mechanisms underlying the transcription of *JUN*. Studies indicate that Chromodomain helicase DNA-binding protein 4 (CHD4) suppresses JUN expression by binding to its promoter region [7], while Hepatic leukemia factor acts a trans-activator of *JUN* [10]. Exopolysaccharides from *Lactobacillus plantarum NCU116* (EPS116) have been shown to enhance both the expression and phosphorylation of JUN [11]. The Nanog homeobox (Nanog) activated JUN transcription through direct binding to its proximal promoter region [12]. The activation of the *JUN* promoter via the CD28 molecule (CD28) was mediated through Myocyte Enhancer Factor 2 [13]. In chickens, circGPD2 (glycerol-3-phosphate dehydrogenase-2-derived circRNA) inhibited JUN by sequestering miR-203a [14]. Nonetheless, the regulatory mechanisms governing the promoter of the chicken *JUN* gene and its role in SSCs remain inadequately understood. Consequently, it is imperative to investigate the activity and regulatory factors associated with the chicken JUN promoter to elucidate the mechanisms underlying its expression regulation and functional roles.

The C-JUN N-terminal kinase (JNK) serves as the primary regulator of JUN phosphorylation and activation. Previous studies have demonstrated that mitogen-activated protein kinase 8, a key gene within the JNK signaling pathway, positively influences the formation of chicken SSC and has an impact on *JUN* expression [15]. The JNK signaling pathway activator, anisomycin, has been observed to enhance JUN transcription in NIH 3T3 cells [16], whereas the JNK signaling pathway inhibitor, sp600125, suppressed JUN expression in esophageal cancer cells [17]. However, there is a lack of studies investigating the regulatory effects of anisomycin and sp600125 on JUN transcription in chickens.

Additionally, Wilms tumor 1 (*WT1*) and CCAAT/enhancer binding protein alpha (*CEBPA*) exhibit differential expression in chicken ESCs and SSCs [8]. CEBPA was linked to the interleukin-1 receptor-associated kinase (IRAK)-1/4/mitogen-activated protein kinase kinase (MKK)/JNK signaling pathways [18], while WT1 was implicated in the regulation of autophagy via the Akt/JNK signaling pathway [19]. The JNK signaling pathway was activated when cells overexpressed cytoma (Rb) inhibitor-associated protein 46, which was upregulated by WT1 [20]. The JNK signaling pathway inhibitor, sp600125, inhibited WT1 protein expression in K562 cells [21] and reversed the downregulation of CEBPA by LPS in 3T3-L1 cells [22]. The regulation of *JUN*, a component of the JNK signaling pathway, by these two transcription factors (TF) remains to be elucidated.

This study examines the activity of the promoter region of *JUN* in chickens and assesses the effects of the JNK signaling pathway activator (anisomycin) and inhibitor (sp600125) on *JUN* transcription. In addition, we investigated the transcriptional regulation of *JUN* expression by transcription factors through cis- and trans-regulation, which are important for studying its regulatory mechanism and role in SSCs.

## 2. Materials and Methods

### 2.1. Materials

The DF-1 cells (chicken embryo fibroblast cell line), pEGFP-N1, pEGFP-linker (pEGFP-N1 with CMV promoter deleted) [23], pRL-SV40, pGL3.0-Basic, OENC (OENC is pcDNA3.1 (+), which is the negative control of two overexpression vectors), OE-WT1 (OE-WT1 is the overexpression vector of *WT1*), and OE-CEBPA (OE-CEBPA is the overexpression vector of *CEBPA*) used in this experiment were stored in the laboratory of the author.

### 2.2. Bioinformatics Analysis of Promoter

The 2000 bp upstream promoter sequences of *JUN* (Gene ID: NM_001031289.2) from the NCBI website were obtained. The region from −2000 bp to 0 bp was analyzed using online prediction tools including BDGP: Neural Network Promoter Prediction [24], Promoter 2.0 Prediction Server [25], FPROM [26], TSSG, TSSP, and TSSW. Based on PROMO HOME PAGE (maximum matrix dissimilarity rate 1%) [27] and JASPAR (relative profile score threshold 85%) predictions, potential TF binding sites in the *JUN* promoter region (−700–0 bp) were identified and analyzed (The URLs of the prediction websites are listed in Table S1).

The 700 bp upstream sequences of *JUN* from chicken and five other species (Table S2) from NCBI were obtained. Using MEGA7, we analyzed homology and constructed a phylogenetic tree.

### 2.3. Promoter Amplification and Vector Construction

Genomic DNA extracted from the testis tissue of 18.5 embryo age Rugao Yellow chicken was used as a template for fragment amplification. Primers used for vector construction were designed by NEBuilder (https://www.neb.com/, accessed on 30 November 2022)), listed in Table S3, and synthesized by Hangzhou Shangya Biological Company. The C1000 TOUCH was used for PCR.

The 2000 bp upstream proximal end of the *JUN* was cloned and used to replace the CMV promoter in pEGFP-N1, constructing the pEGFP-JUN-promoter. Removing the CMV promoter and linearizing pEGFP-N1 were achieved with AseI (R0526V, New England Biolabs, Beverly, MA, USA) and BamHI (R3136V, New England Biolabs, Beverly, MA, USA). The unidirectional deletions of *JUN*’s promoters were designed using the promoter prediction results to construct pGL3-P1, pGL3-P2, and pGL3-P3.

To identify the binding sites for CEBPA and WT1 on the *JUN* promoter, deletion vectors for the CEBPA and WT1 sites were conducted for the site-deletion analysis. Using pGL3-P1 as a template, the corresponding primers in Table S3 were amplified and the corresponding promoter fragments were ligated to pGL3-Basic linearized by KpnI (1618, TaKaRa, Tokyo, Japan) and HindIII (1615, TaKaRa, Tokyo, Japan).

All fragments were amplified by PrimeSTAR^®^ Max DNA Polymerase (R045A, TaKaRa, Tokyo, Japan), purified with a DNA purification kit (DC301, Vazyme, Shanghai, China), and then ligated into the linearized vectors using the ClonExpress Ultra One Step Cloning Kit (C115-02, Vazyme, Shanghai, China). For sequencing, recombinant plasmids were sent to Hangzhou Shangya Biological Company.

### 2.4. Cell Culture and Treatment

In the culture of the DF-1 cells, Dulbecco’s Modified Eagle Medium (DMEM) (c11995500BT, Gibco, NY, USA) containing 10% fetal bovine serum (FBS) (900-108, Gemini, Calabasas, CA, USA) was used.

DF-1 cells were seeded into 12-well plates at a density of 5 × 10^5^ cells per well. When the cells reached approximately 60% confluence, they were treated with 10 μM Anisomycin (HY-18982, MedChemExpress, Monmouth Junction, NJ, USA) and 20 μM Sp600125 (HY-12041, MedChemExpress, Monmouth Junction, NJ, USA) or transfected with plasmids at a ratio of 3 μL Exfect to 1 μg plasmid DNA using Exfect^®^ Transfection Reagent (T101-01, Vazyme, Shanghai, China) following manufacturer’s instruction. After treatment, qRT-PCR was used to detect the expression of related genes.

DF-1 cells were seeded into 24-well plates. At approximately 60% confluence, they were co-transfected with 14 ng pRL-SV40 and 500 ng of pGL3 recombinant plasmid using Exfect^®^ Transfection Reagent. A negative control group was also set up (combining pGL3-basic and pRL-SV40 transfections). Cells were co-transfected with14 ng of pRL-SV40, 500 ng of pGL3 recombinant plasmid, and 500 ng of OE-WT1/OE-CEBPA. A negative control group was also set up (co-transfection of pRL-SV40, pGL3-basic, and OENC). After 48 h post-transfection, cells were collected for dual-luciferase activity assays. At least three repetitions were performed for each reporter assay.

### 2.5. qRT-PCR

According to the manufacturer’s protocol, total RNA was extracted from test samples using RNAiso Plus (9109, TaKaRa, Tokyo, Japan) and reverse-transcribed using a PrimeScript™ RT Reagent Kit with gDNA Eraser (RR047A, TaKaRa, Tokyo, Japan). The C1000 TOUCH was used for reverse transcription. A NovoStart R SYBR qPCR SuperMix Plus (E096-01A, Novoprotein, Shanghai, China) was used to prepare the PCR mixture. The CFX-96TOUCH was used for qRT-PCR detection. The running program was 95 °C for 1 min (initial denaturation), followed by 95 °C for 20 s, 60 °C for 20 s, and 72 °C for 30 s, for a total of 40 cycles. Relative gene expression after normalization is compared against chicken *B-ACTIN* expression as an endogenous control. Primers for qRT-PCR were listed in Table 1 and synthesized by Hangzhou Shangya Biological Company. Data analysis was conducted using the 2^−ΔΔCt^ method.

### 2.6. Dual-Luciferase Assay

The Dual Luciferase Reporter Kit (DL101-01, Vazyme, Shanghai, China) was used to measure the relative luciferase activity of test samples 48 h after transfection. The specific steps were as follows: PBS was used to wash the cells after discarding the original culture medium. Cells were lysed by adding 100 µL of 1×cell lysis buffer to each well and shaking at room temperature for 5 min. After transferring the lysate to a 1.5 mL centrifuge tube, centrifugation at 11,200 rpm for two minutes at room temperature was performed. In another centrifuge tube, 10 µL of the supernatant was mixed with 50 µL of room-temperature equilibrated Luciferase Substrate. Firefly luciferase activity was immediately measured using a Promega Glomax luminometer(Promega, Madison, WI, USA). The luciferase activity of the Renilla substrate was then measured immediately after adding 50 µL of freshly prepared working solution to the reaction mixture. At least three repetitions were performed for each reporter assay.

### 2.7. Statistical Analysis

SPSS 19.0 was used to process all data. Among the two groups, an independent sample t-test was appropriate, while a one-way analysis of variance was appropriate for comparisons among several groups. Results were defined as significant at *p* < 0.05 and extreme significance at *p* < 0.01. The histograms were generated using GraphPad Prism 6.

## 3. Results

### 3.1. Analysis of the JUN Promoter

**Figure 1:**
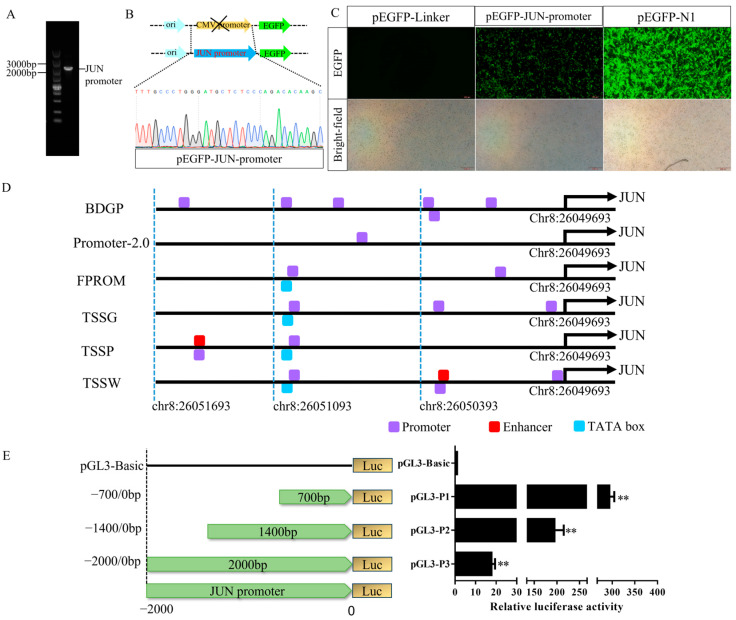
Analysis of the JUN promoter. (**A**) Amplification of the full-length JUN promoter (including homologous arms with pEGFP-N1). (**B**) Schematic diagram and chromatograms of the pEGFP-JUN-promoter vector. (**C**) Transfection of pEGFP-JUN-promoter into DF-1 cells, qualitative analysis of JUN promoter activity as indicated by green fluorescence. (**D**) Promoter sequence elements of the upstream 2000 bp JUN sequence predicted by various online tools. (**E**) Schematic diagram and dual-luciferase assay results of different truncated JUN promoter constructs. Note: pGL3-P1: Fragment 1 (−700 to 0 bp, chr8:26050393-26049693) cloned into the pGL3-Basic vector; pGL3-P2: Fragment 2 (−1400 to 0 bp, chr8:26051093-26049693) cloned into the pGL3-Basic vector; pGL3-P3: Fragment 3 (−2000 to 0 bp, chr8:26051693-26049693) cloned into the pGL3-Basic vector; *n* = 3, **: *p* < 0.01.

To gain a better understanding of the transcriptional regulation of the *JUN* gene, we replaced the CMV promoter in pEGFP-N1 with 2000 bp upstream of *JUN* (Figure 1A,B). Then, DF-1 cells were transfected with it, and 48 h later, green fluorescence was observed. Results showed that the fluorescence of pEGFP-JUN-promoter was weaker than that of pEGFP-N1 and stronger than that of pEGFP-Linker (the vector without CMV promoter) (Figure 1C), indicating that the −2000 bp–0 bp sequence has promoter activity.

To detect the activity of different fragments in the upstream region of *JUN*, three 5′ deletion luciferase reporter vectors based on the online prediction results of the −2000–0 bp sequence were constructed (Figure 1D,E). DF-1 cells were transfected with them and their dual-luciferase activity was measured (Figure 1E). The results showed that the pGL3-P1 (−700–0 bp) luciferase activity was the highest and significantly higher than pGL3-P2 (−1400–0 bp) and pGL3-P3 (−2000–0 bp).

### 3.2. Anisomycin Activates JUN Transcription

**Figure 2:**
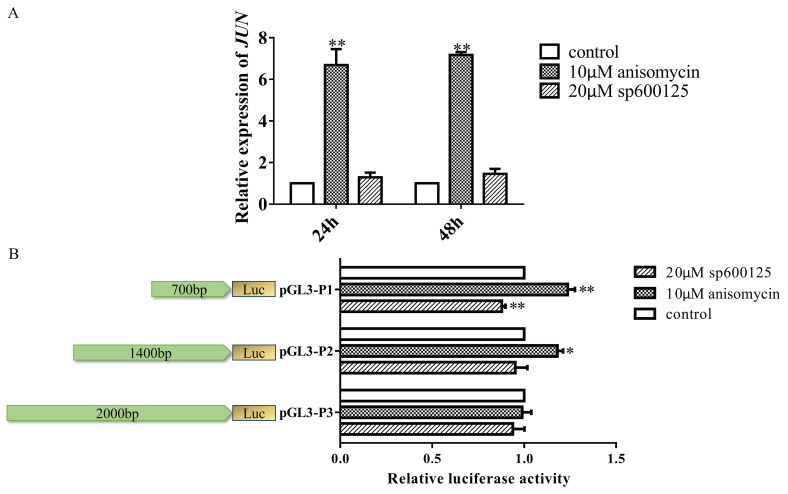
JNK signaling pathway activator activates JUN transcription. (**A**) JUN expression changes in DF-1 cells treated with the JNK signaling pathway activator (10 μM anisomycin) and inhibitor (20 μM sp600125) were analyzed using qRT-PCR. (**B**) After transfecting the recombinant vectors for 24 h, the cells were treated with the JNK signaling pathway activator (10 μM anisomycin) and inhibitor (20 μM sp600125) for an additional 24 h. Relative luciferase activity of each vector was measured using the dual-luciferase reporter assay. *n* = 3, *: *p* < 0.05, **: *p* < 0.01.

To clarify the regulation of *JUN* transcription by JNK pathway, DF-1 cells were treated with 10 μM anisomycin (activator of the JNK signaling pathway) and 20 μM sp600125 (inhibitor of the JNK signaling pathway), and *JUN* mRNA expression was detected by qRT-PCR (Figure 2A). With anisomycin treatment, *JUN* expression was significantly upregulated compared to the control group, while with sp600125 treatment, no significant change was observed. We also measured the mRNA expression of the JNK signaling molecules *MAPK8* and *ATF2* in DF-1 cells treated with JNK signaling pathway activator/inhibitor, and found that the expression of both was up-regulated after anisomycin treatment, and the expression of *MAPK8* was down-regulated after sp600125 treatment, but there was no differential change in *ATF2* (Figure S1).

Additionally, the pGL3 constructs were treated with anisomycin and sp600125 (Figure 2B). We found that anisomycin significantly increased the luciferase activity of pGL3-P1 and pGL3-P2, but not pGL3-P3. The luciferase activity of pGL3-P1 significantly decreased after treatment with sp600125, while that of pGL3-P2 and pGL3-P3 decreased, but not significantly.

### 3.3. Bioinformatics Analysis of −700–0 bp of the JUN Promoter

**Figure 3:**
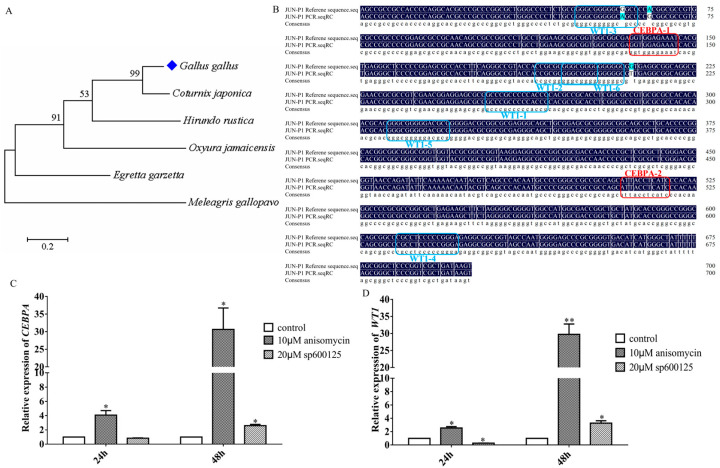
Bioinformatics analysis of −700–0 bp of the JUN promoter. (**A**) Phylogenetic analysis of the −700 to 0 bp upstream region of JUN in different species. (**B**) Predicted transcription factor binding sites in the −700 to 0 bp region upstream of the chicken JUN transcription start site, with WT1 and CEBPA being the primary factors. The suffix numbers indicate predicted scores, decreasing from 1 to 6. (**C**,**D**) Expression changes of CEBPA and WT1 in DF-1 cells after treatment with the JNK signaling pathway activator (10 μM anisomycin) and inhibitor (20 μM sp600125), as detected by qRT-PCR. *n* = 3, *: *p* < 0.05, **: *p* < 0.01.

Based on promoter activity and treatment of the JNK signaling pathway activator and inhibitor, we focused on pGL3-P1. To determine the conservation of *JUN* (−700–0 bp) in chicken, MEGA7 was applied to analyze homology of upstream 700 bp sequences of *JUN* in chicken and other species and draw the phylogenetic tree. It was found that the upstream −700–0 bp of *JUN* in chicken (Gallus gallus) and Japanese quail (Coturnix japonica) was more conserved and similar to other birds (Figure 3A and Figure S2).

To identify potential transcription factors regulating *JUN* transcription, we utilized PROMO and JASPAR to predict transcription factor binding sites within the *JUN* (−700–0 bp) region, and integrated these predictions with sequencing data from Zhang [8]. Transcription factors WT1 and CEBPA were predicted and screened (Figure 3B). A significant increase in *WT1* and *CEBPA* expression was observed after the treatment of anisomycin. *WT1* was significantly inhibited by sp600125 for 24 h, but *CEBPA* was not affected, and after 48 h both were upregulated (Figure 3C,D).

### 3.4. Binding Sites of WT1 and CEBPA Affect JUN Core Promoter Activity

**Figure 4:**
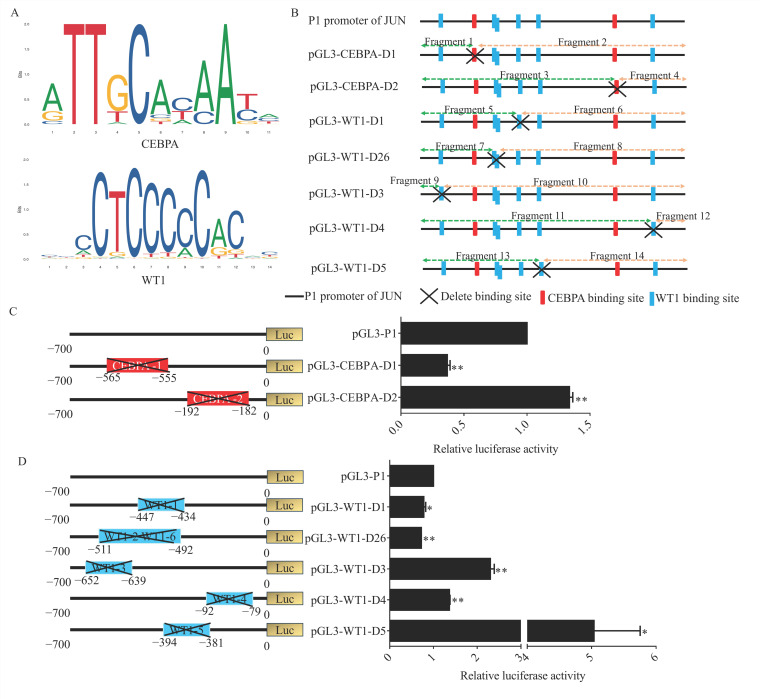
Validation of the effects of WT1 and CEBPA binding sites on JUN promoter activity. (**A**) Predicted binding sites of WT1 and CEBPA on the JUN promoter region. (**B**) Schematic of the construction of WT1 and CEBPA binding site deletion vectors. (**C**,**D**) Changes in JUN promoter activity following the deletion of WT1 and CEBPA binding sites, assessed using a dual-luciferase reporter system. *n* = 3, *: *p* < 0.05, **: *p* < 0.01.

To further verify the effects of WT1 and CEBPA binding sites (Figure 4A) on the *JUN* promoter, deletion vectors lacking the WT1 and CEBPA binding sites were constructed (Figure 4B and Figure S3), respectively. *JUN* promoter activity was significantly altered when WT1 and CEBPA binding sites were deleted (Figure 4C,D). Upon deletion of CEBPA binding sites 1 and WT1 binding sites 1, 2 and 6, activity of −700–0 bp of the *JUN* promoter was inhibited. When CEBPA binding sites 2 and WT1 binding sites 3, 4, and 5 were deleted, the activity of −700–0 bp of the *JUN* promoter increased.

### 3.5. WT1 Repressed JUN Transcription

**Figure 5:**
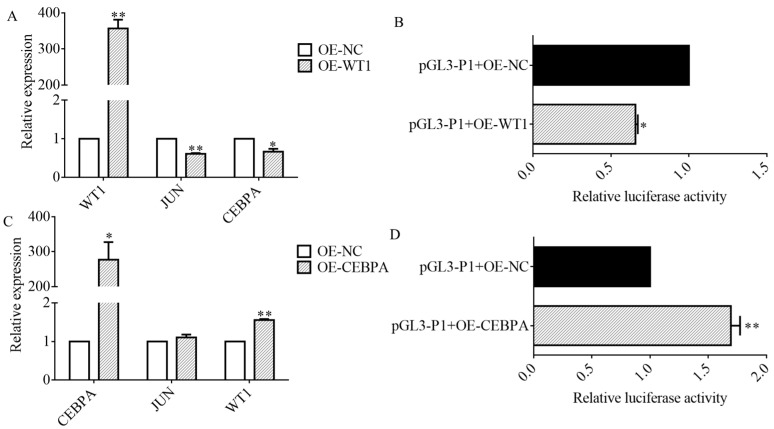
WT1 and CEBPA affected JUN transcription. (**A**) After overexpression of WT1, mRNA levels of WT1, JUN, and CEBPA were detected by qRT-PCR. (**B**) After overexpression of WT1, relative luciferase activity of pGL3-P1 was detected by dual-luciferase reporter assay. (**C**) After overexpression of CEBPA, mRNA levels of CEBPA, JUN, and WT1 were detected by qRT-PCR. (**D**) After overexpression of CEBPA, relative luciferase activity of pGL3-P1 was detected by dual-luciferase reporter assay. *n* = 3, *: *p* < 0.05, **: *p* < 0.01.

To determine the effect of WT1 on *JUN*, the mRNA level of *JUN* was detected after *WT1* overexpression, and the results showed that the expression of *JUN* and *CEBPA* decreased significantly after *WT1* overexpression (Figure 5A). Furthermore, we co-transfected pGL3-P1 with OE-WT1, detected pGL3-P1 luciferase activity after 48 h, and found that *WT1* overexpression significantly inhibited pGL3-P1 luciferase activity (Figure 5B). We also co-transfected OE-WT1 with pGL3 vectors with a deletion of WT1 binding sites and found that overexpression of *WT1* significantly inhibited their relative luciferase activity (Figure S4).

To ascertain the effect of CEBPA on *JUN*, *CEBPA* was overexpressed. It was found that the mRNA level of *JUN* increased, but not significantly, while the expression of *WT1* was significantly upregulated (Figure 5C). We also co-transfected pGL3-P1 with OE-CEBPA into DF-1 and found that the luciferase activity of pGL3-P1 increased significantly (Figure 5D). With OE-CEBPA co-transfected with pGL3 vector without CEBPA binding sites, we found that CEBPA did not affect the relative luciferase activity of the promoter when binding site 1 was deleted, whereas it increased activity when binding site 2 was deleted (Figure S5).

## 4. Discussion

Here, we identified the chicken *JUN* promoter and found that the JNK signaling pathway activator anisomycin activated *JUN* transcription while promoting the expression of the transcription factors *WT1* and *CEBPA*. We further discovered that the binding sites of transcription factors *WT1* and *CEBPA* regulate the −700–0 bp promoter activity of *JUN*, with *WT1* inhibiting *JUN* transcription and *CEBPA* participating in its regulation. This study lays the groundwork for future research on the role and regulatory mechanisms of JUN in the formation of spermatogonial stem cells (SSCs).

As a member of the activating protein 1 (AP-1) family, JUN plays an important role in several cellular processes, including cell renewal, proliferation, and apoptosis [28,29]. It was found that *JUN* was highly expressed in mouse type B spermatogonia [30] and chicken SSCs [8]. Overexpression of *MAPK8* could up-regulate the expression of chicken SSC marker genes and *JUN* [15]. JUN could regulate miRNA transcription related to chicken SSC differentiation [9]. T-2 promoted the apoptosis of GC-1 cells, induced the phosphorylation of JUN, JNK/SAPK, and up-regulated the expression of genes related to the JNK signaling pathway [31]. These results indicate that *JUN* is closely related to the growth of spermatogonia. The knockout of JUN could promote the survival of mouse mammary epithelial cells and induce the expression of apoptosis genes [32], and also improve the induction efficiency of human ESC cardiomyocytes [33]. Overexpression of JUN could inhibit normal cardiomyocyte morphology and severely inhibit the production of TNNT2+ cells [33]. Therefore, the correct expression of *JUN* plays an important role in the body. *JUN* can be regulated by various extracellular signals, such as growth factors, cytokines, and extracellular stress, usually through JNK [34,35,36]. However, there are limited reports on the transcriptional regulation mechanisms of *JUN*. The transcriptional regulation of genes is affected by the activity of different transcription sites in the promoter region. Transcription factors (TFs) can specifically recognize the active region of a gene promoter, thereby activating or inhibiting the transcriptional activity of a target gene, and regulating the mRNA transcription level of the gene [37]. At present, the molecular mechanism of gene transcription in chicken *JUN* is still unclear. To this end, we predicted and analyzed the 2000 bp sequence upstream of the transcription start site of chicken *JUN* and confirmed the priming activity of this region by using the intensity of GFP fluorescence. Then, three pGL3 recombinant vectors containing different lengths were constructed, and the initiation activity of each fragment in the *JUN* promoter region was confirmed by using a dual luciferase reporter system. The activation effect of the JNK signaling pathway activator on *JUN* was confirmed, and the binding sites of WT1 and CEBPA were predicted in the 700 bp region upstream of *JUN*.

JUN, a crucial component of the JNK signaling pathway, functions as a transcription factor that regulates various physiological processes. The transcription of *JUN* was promoted by the JNK signaling pathway activator anisomycin in NIH 3T3 cells [16].In our research, anisomycin activated chicken *JUN* transcription. We also found that the JNK signaling pathway inhibitor sp600125 only inhibited the luciferase activity of pGL3-P1 and had no obvious effect on pGL3-P2, pGL3-P3, and the mRNA expression of *JUN*. Ban reported that sp600125 inhibited the phosphorylation of JUN [38]. Therefore, it is speculated that sp600125 does not affect the transcription of chicken *JUN* and may affect its activity in other ways.

A zinc finger transcription factor, Wilms tumor gene 1 (WT1) contributes to the development and function of the testicles [39,40,41,42]. Zhang et al. found differential expression of *WT1* in chicken embryonic stem cells and chicken SSCs [8]. We discovered that WT1 bound to the *JUN* promoter region and inhibited *JUN* transcription. Therefore, we hypothesize that *WT1* is likely involved in SSC physiology through JUN. We also found that the anisomycin upregulated *WT1* expression, consistent with Anuchapreeda et al., who reported that JNK/JUN signaling induced *WT1* gene expression [21]. Anuchapreeda also found that knocking out *JUN* could inhibit *WT1* expression. In contrast, we found that *WT1* inhibited *JUN* transcription. Collectively, these findings suggest that the regulation of *JUN* by WT1 is independent on JNK signaling activation by anisomycin. It is likely that *WT1* and *JUN* mutually regulate each other, with *WT1*’s effect on *JUN* being possibly weaker than anisomycin’s effect on *JUN*.

The CCAAT/enhancer binding protein alpha (CEBPA) plays an essential role in the transduction of transforming growth factor-activated kinases-1 (TAK1) and JNK signaling pathways [43]. JUN changes often coincide with CEBPA changes [44]. We found that chicken *JUN* promoter regions contained CEBPA binding sites, and deletion of these sites affected pGL3-P1 promoter activity. The relative luciferase activity of pGL3-P1 increased significantly after *CEBPA* overexpression, and the *JUN* also increased accordingly, but it did not show a significant difference. Meanwhile, WT1 expression was significantly up-regulated during this process. We also found that when WT1 expression was increased, the mRNA level of CEBPA decreased. Wang et al. reported that mutations in *CEBPA* in adult acute myeloid leukemia often co-occur with mutations in *WT1* [45]. Therefore, it is speculated that in addition to the direct binding to the *JUN* promoter region for regulation, it is very likely that *CEBPA* and *WT1* have negative feedback modulation, which jointly affects the expression of *JUN*. These need to be proved by experiments in the future.

Interestingly, we found that the expression of *WT1* and *CEBPA* was down-regulated at 24 h and up-regulated at 48 h after JNK signaling pathway inhibitor treatment. We speculated that JNK signaling pathway had a positive regulatory effect on *WT1* and *CEBPA* expression at the beginning of sp600125 treatment, but over time, cells may activate other stress-related signaling pathways through negative feedback mechanisms, which may in turn promote the up-regulation of their expression. *WT1* and *CBEPA* play an important role in cell proliferation [46,47], differentiation [48,49], and apoptosis [50,51], so it is also possible for cells to ensure that their expression levels are restored or enhanced through compensatory mechanisms [52], but these will require further exploration at a later stage.

## 5. Conclusions

This study identified that the −700–0 bp upstream of the transcription start site of *JUN* had the highest promoter activity. We showed that the JNK signaling pathway activator anisomycin activated *JUN* transcription and upregulated the expression of the transcription factors *WT1* and *CEBPA*. *WT1* suppressed the transcription of *JUN*, while *CEBPA* promoted the transcription of *JUN*. Collectively, these findings aid understanding of transcriptional regulation of the *JUN* in chicken.

## Figures and Tables

**Table 1:** qRT-PCR primer sequences of related genes.

Gene	Gene ID	Primer (5′-3′) of qRT-PCR	Tm (°C)
*B-ACTIN*	NM_205518.2	qF: ACCAACTGGGATGATATGGAGAA	60
		qR:TTGGCTTTGGGGTTCAGG	
*JUN*	NM_001031289.2	qF: CTCATCATCCAGTCCAGCAA	60
		qR: TGTTCTGGTTGTGCAGTTCC	
*CEBPA*	NM_001031459.2	qF: TCGGCGACATCTGCGAGAAC	60
		qR: TGCTTGCTGTGCTGGAAGAGG	
*WT1*	NM_001397547.1	qF: GCCCCTTCATGTGTGCCTACC	60
		qR: GTGTCGTCTTTGGTGCCGTTTC	
*MAPK8*	XM_046942979.1	qF: CAGCCAGCCCCTTTAGGTG	60
		qR: CTACAGCAACCCAGAGGTCCAG	
*ATF2*	NM_204904.2	qF: CCAGGCTCCATCCTCTAACA	60
		qR: AGGAATAGCAACAGGCATGG	

## Data Availability

The original data in the article can be obtained directly from the first author. The data are not publicly available due to privacy restrictions.

## Supplementary Materials

The following supporting information can be downloaded at: https://www.mdpi.com/article/10.3390/genes15101351/s1, Figure S1: Analysis of JNK signaling molecules (*MAPK8* and *ATF2*) in DF-1 cells treated with anisomycin and sp600125. *: *p* < 0.05, **: *p* < 0.01. Figure S2: Alignment of −700–0 bp sequences upstream of *JUN* transcription start site between chicken (Gallus gallus) and Japanese quail (Coturnix japonica). Figure S3: Sequencing and alignment of luciferase recombinant plasmids missing the WT1 binding sites and the CEBPA binding sites. Figure S4: Effect of overexpression of *WT1* on the relative luciferase activity of pGL3 recombinant vectors with deletion of WT1 binding site. *: *p* < 0.05, **: *p* < 0.01. Figure S5: Effect of overexpression of *CEBPA* on the relative luciferase activity of pGL3 recombinant vectors with deletion of CEBPA binding site. *: *p* < 0.05, **: *p* < 0.01. Table S1: Online prediction software predicts URLs. Table S2: Information on the *JUN* promoter for 6 species. Table S3: lists all primers used for the plasmid construction.

## References

[1] Hussein M.S., Li Q., Mao R., Peng Y., He Y. (2023). TCR T cells overexpressing c-Jun have better functionality with improved tumor infiltration and persistence in hepatocellular carcinoma. Front. Immunol..

[2] Zhao X., Bartholdy B., Yamamoto Y., Evans E.K., Alberich-Jorda M., Staber P.B., Benoukraf T., Zhang P., Zhang J., Trinh B.Q. (2022). PU.1-c-Jun interaction is crucial for PU.1 function in myeloid development. Commun. Biol..

[3] Wang G., Zhao Z., Ren B., Yu W., Zhang X., Liu J., Wang L., Si D., Yang M. (2022). Exenatide exerts a neuroprotective effect against diabetic cognitive impairment in rats by inhibiting apoptosis: Role of the JNK/c-JUN signaling pathway. Mol. Med. Rep..

[4] Alcivar A.A., Hake L.E., Hardy M.P., Hecht N.B. (1990). Increased levels of junB and c-jun mRNAs in male germ cells following testicular cell dissociation. Maximal stimulation in prepuberal animals. J. Biol. Chem..

[5] Suomalainen L., Dunkel L., Ketola I., Eriksson M., Erkkila K., Oksjoki R., Taari K., Heikinheimo M., Pentikainen V. (2004). Activator protein-1 in human male germ cell apoptosis. Mol. Hum. Reprod..

[6] Zhang Y., Su Y.L., Li L.S., Yang Z., Chen S., Xiong J., Fu X.H., Peng X.N. (2015). Mouse dead end 1-beta interacts with c-Jun and stimulates activator protein 1 transactivation. Mol. Med. Rep..

[7] Li P., Tang J., Yu Z., Jin C., Wang Z., Li M., Zou D., Mang X., Liu J., Lu Y. (2022). CHD4 acts as a critical regulator in the survival of spermatogonial stem cells in micedagger. Biol. Reprod..

[8] Zhang Z., Elsayed A.K., Shi Q., Zhang Y., Zuo Q., Li D., Lian C., Tang B., Xiao T., Xu Q. (2015). Crucial genes and pathways in chicken germ stem cell differentiation. J. Biol. Chem..

[9] Wang Y., Kong R., Xie K., Hu C., Zhao Z., Wu Y., Zuo Q., Li B., Zhang Y. (2023). Analysis of the Promoter Regions of gga-miR-31 and Its Regulation by RA and C-jun in Chicken. Int. J. Mol. Sci..

[10] Xiang D.M., Sun W., Zhou T., Zhang C., Cheng Z., Li S.C., Jiang W., Wang R., Fu G., Cui X. (2019). Oncofetal HLF transactivates c-Jun to promote hepatocellular carcinoma development and sorafenib resistance. Gut.

[11] Zhou X., Zhang K., Qi W., Zhou Y., Hong T., Xiong T., Xie M., Nie S. (2019). Exopolysaccharides from Lactobacillus plantarum NCU116 Enhances Colonic Mucosal Homeostasis by Controlling Epithelial Cell Differentiation and c-Jun/Muc2 Signaling. J. Agric. Food. Chem..

[12] Lin Y., Xiong F., Zhou Y., Wu X., Liu F., Xue S., Chen H. (2015). NANOG upregulates c-Jun oncogene expression through binding the c-Jun promoter. Mol. Carcinog..

[13] Shin H.M., Han T.H. (1999). CD28-mediated regulation of the c-jun promoter involves the MEF2 transcription factor in Jurkat T cells. Mol. Immunol..

[14] Shen X., Cui C., Tang S., Han S., Zhang Y., Xia L., Tan B., Ma M., Kang H., Yu J. (2022). MyoG-enhanced circGPD2 regulates chicken skeletal muscle development by targeting miR-203a. Int. J. Biol. Macromol..

[15] Wang Y., Bi Y., Zuo Q., Zhang W., Li D., He N.N., Cheng S., Zhang Y.N., Li B. (2018). MAPK8 regulates chicken male germ cell differentiation through JNK signaling pathway. J. Cell. Biochem..

[16] Marinissen M.J., Chiariello M., Tanos T., Bernard O., Narumiya S., Gutkind J.S. (2004). The small GTP-binding protein RhoA regulates c-jun by a ROCK-JNK signaling axis. Mol. Cell.

[17] Yu X., Luo A., Zhou C., Ding F., Wu M., Zhan Q., Liu Z. (2006). Differentiation-associated genes regulated by TPA-induced c-Jun expression via a PKC/JNK pathway in KYSE450 cells. Biochem. Biophys. Res. Commun..

[18] Bhargavan B., Kanmogne G.D. (2018). Differential Mechanisms of Inflammation and Endothelial Dysfunction by HIV-1 Subtype-B and Recombinant CRF02_AG Tat Proteins on Human Brain Microvascular Endothelial Cells: Implications for Viral Neuropathogenesis. Mol. Neurobiol..

[19] Mo H., He J., Yuan Z., Mo L., Wu Z., Lin X., Liu B., Guan J. (2016). WT1 is involved in the Akt-JNK pathway dependent autophagy through directly regulating Gas1 expression in human osteosarcoma cells. Biochem. Biophys. Res. Commun..

[20] Li G.C., Guan L.S., Wang Z.Y. (2003). Overexpression of RbAp46 facilitates stress-induced apoptosis and suppresses tumorigenicity of neoplastigenic breast epithelial cells. Int. J. Cancer..

[21] Anuchapreeda S., Rungrojsakul M., Tima S., Chiampanichayakul S., Krig S.R. (2019). Co-activation of WT1 and AP-1 proteins on WT1 gene promoter to induce WT1 gene expression in K562 cells. Cell Signal..

[22] Xiang X., An W., Jiang C., Zhao J., Wang X., Sun G., Li Y., Zhang W. (2013). Lipopolysaccharide inhibits the expression of resistin in adipocytes. J. Mol. Endocrinol..

[23] Wang H., Zhang L., Hou M., Wang C., Wang L., Sun T., He H., Zhong J. (2011). Cloning and Activity Analysis of Bovine Natural Resistance Associated Macrophage Protein 1 (Nramp1) Gene Promoter. Sci. Agric. Sin..

[24] Khan R., Ali Z., Niazi A.K., Carolan J.C., Wilkinson T.L. (2020). In silico Characterization of a Candidate Protein from Aphid Gelling Saliva with Potential for Aphid Control in Plants. Protein Pept. Lett..

[25] Chen Y., He X., Cheng F., Li M., Wu X., Zhang C., Li J., Huang B., Qi M. (2021). Angiotensin II promotes EMT of hepatocellular carcinoma cells through high mobility group protein B1 mediated by E4F1. Biochem. Biophys. Res. Commun..

[26] Solovyev V., Kosarev P., Seledsov I., Vorobyev D. (2006). Automatic annotation of eukaryotic genes, pseudogenes and promoters. Genome Biol..

[27] Liu X., Chen J.G., Munshi M., Hunter Z.R., Xu L., Kofides A., Tsakmaklis N., Demos M.G., Guerrera M.L., Chan G.G. (2020). Expression of the prosurvival kinase HCK requires PAX5 and mutated MYD88 signaling in MYD88-driven B-cell lymphomas. Blood Adv..

[28] Angel P., Karin M. (1991). The role of Jun, Fos and the AP-1 complex in cell-proliferation and transformation. Biochim. Biophys. Acta.

[29] Kovary K., Bravo R. (1991). The jun and fos protein families are both required for cell cycle progression in fibroblasts. Mol. Cell. Biol..

[30] Alcivar A.A., Hake L.E., Kwon Y.K., Hecht N.B. (1991). junD mRNA expression differs from c-jun and junB mRNA expression during male germinal cell differentiation. Mol. Reprod. Dev..

[31] Lee W.Y., Park H.J. (2023). T-2 mycotoxin Induces male germ cell apoptosis by ROS-mediated JNK/p38 MAPK pathway. Ecotoxicol. Environ. Saf..

[32] Katiyar S., Casimiro M.C., Dettin L., Ju X., Wagner E.F., Tanaka H., Pestell R.G. (2010). C-jun inhibits mammary apoptosis in vivo. Mol. Biol. Cell.

[33] Zhong H., Zhang R., Li G., Huang P., Zhang Y., Zhu J., Kuang J., Hutchins A.P., Qin D., Zhu P. (2023). c-JUN is a barrier in hESC to cardiomyocyte transition. Life Sci. Alliance.

[34] Davis R.J. (2000). Signal transduction by the JNK group of MAP kinases. Cell.

[35] Liu Y., Long Y., Xing Z., Zhang D. (2015). C-Jun recruits the NSL complex to regulate its target gene expression by modulating H4K16 acetylation and promoting the release of the repressive NuRD complex. Oncotarget.

[36] Yung J., Giacca A. (2020). Role of c-Jun N-terminal Kinase (JNK) in Obesity and Type 2 Diabetes. Cells.

[37] Yang L., Orenstein Y., Jolma A., Yin Y., Taipale J., Shamir R., Rohs R. (2017). Transcription factor family-specific DNA shape readout revealed by quantitative specificity models. Mol. Syst. Biol..

[38] Ban K., Santora R., Kozar R.A. (2011). Enteral arginine modulates inhibition of AP-1/c-Jun by SP600125 in the postischemic gut. Mol. Cell. Biochem..

[39] Natoli T.A., Alberta J.A., Bortvin A., Taglienti M.E., Menke D.B., Loring J., Jaenisch R., Page D.C., Housman D.E., Kreidberg J.A. (2004). Wt1 functions in the development of germ cells in addition to somatic cell lineages of the testis. Dev. Biol..

[40] Rao M.K., Pham J., Imam J.S., Maclean J.A., Murali D., Furuta Y., Sinha-Hikim A.P., Wilkinson M.F. (2006). Tissue-specific RNAi reveals that WT1 expression in nurse cells controls germ cell survival and spermatogenesis. Genes Dev..

[41] Wang X.N., Li Z.S., Ren Y., Jiang T., Wang Y.Q., Chen M., Zhang J., Hao J.X., Wang Y.B., Sha R.N. (2013). The Wilms tumor gene, Wt1, is critical for mouse spermatogenesis via regulation of sertoli cell polarity and is associated with non-obstructive azoospermia in humans. PLoS Genet..

[42] Boublikova L., Bakardjieva-Mihaylova V., Skvarova K.K., Kuzilkova D., Dobiasova A., Fiser K., Stuchly J., Kotrova M., Buchler T., Dusek P. (2016). Wilms tumor gene 1 (WT1), TP53, RAS/BRAF and KIT aberrations in testicular germ cell tumors. Cancer Lett..

[43] Bhargavan B., Kanmogne G.D. (2018). Toll-Like Receptor-3 Mediates HIV-1-Induced Interleukin-6 Expression in the Human Brain Endothelium via TAK1 and JNK Pathways: Implications for Viral Neuropathogenesis. Mol. Neurobiol..

[44] Bhargavan B., Woollard S.M., Kanmogne G.D. (2016). Toll-like receptor-3 mediates HIV-1 transactivation via NFkappaB and JNK pathways and histone acetylation, but prolonged activation suppresses Tat and HIV-1 replication. Cell. Signal..

[45] Wang T., Hua H., Wang Z., Wang B., Cao L., Qin W., Wu P., Cai X., Chao H., Lu X. (2022). Frequency and clinical impact of WT1 mutations in the context of CEBPA-mutated acute myeloid leukemia. Hematology.

[46] Yao Y., Chai X., Gong C., Zou L. (2021). WT1 inhibits AML cell proliferation in a p53-dependent manner. Cell Cycle.

[47] Ellsworth P.N., Herring J.A., Leifer A.H., Ray J.D., Elison W.S., Poulson P.D., Crabtree J.E., Van Ry P.M., Tessem J.S. (2024). CEBPA Overexpression Enhances beta-Cell Proliferation and Survival. Biology.

[48] Guo Z., Sun L., Xia H., Tian S., Liu M., Hou J., Li J., Lin H., Du G. (2022). Shikonin as a WT1 Inhibitor Promotes Promyeloid Leukemia Cell Differentiation. Molecules.

[49] Kantzer C.G., Yang W., Grommisch D., Vikhe P.K., Mak K.H., Shirokova V., Genander M. (2022). ID1 and CEBPA coordinate epidermal progenitor cell differentiation. Development.

[50] Lv L., Chen G., Zhou J., Li J., Gong J. (2015). WT1-AS promotes cell apoptosis in hepatocellular carcinoma through down-regulating of WT1. J. Exp. Clin. Cancer Res..

[51] Su R., Dong L., Li C., Nachtergaele S., Wunderlich M., Qing Y., Deng X., Wang Y., Weng X., Hu C. (2018). R-2HG Exhibits Anti-tumor Activity by Targeting FTO/m(6)A/MYC/CEBPA Signaling. Cell.

[52] Huang R., Zhang X., Gracia-Sancho J., Xie W.F. (2022). Liver regeneration: Cellular origin and molecular mechanisms. Liver Int..

