# Supplementary material for: A Study of JUN’s Promoter Region and Its Regulators in Chickens
Source: Genes (Basel). 2024 Oct 21;15(10):1351. doi: 10.3390/genes15101351 (PMC11508107; doi:10.3390/genes15101351)
Supplement: Supplementary file 1 [file genes-15-01351-s001.zip › Supplementary Materials-Tables S1-S3.pdf]

Table S1 Online prediction software predicts URLs

The name of the online prediction software	website
NCBI	https://www.ncbi.nlm.nih.gov/
BDGP	http://www.fruitfly.org/seq_tools/promoter.html
Promoter 2.0	http://www.cbs.dtu.dk/servic-es/Promoter/
FPROM	http://www.softberry.com/berry.phtml?topic=fprom&group=programs&subgroup=promoter
TSSG	http://www.softberry.com/berry.phtml?topic=tssg&group=programs&subgroup=promoter
TSSP	http://www.softberry.com/berry.phtml?topic=tssp&group=programs&subgroup=promoter
TSSW	http://www.softberry.com/berry.phtml?topic=tssw&group=programs&subgroup=promoter
PROMO HOME PAGE	http://alg gen.lsi. upc.es/cgi-bin/promo_v3/promo/prom oinit.cgi?dirDB=TF_8.3
JASPAR	https://jaspar.elixir.no/

Table S2 Information on the *JUN* promoter for 6 species

Species	Gene ID	Location
Chicken（*Gallus gallus*）	NM_001031289.2	Chr8: 26050393-26049693
Turkey(*Meleagris gallopavo*)	XM_003208888.4	Chr10:20961535-20960835
Little egret (*Egretta garzetta*)	XM_009636238.2	6459213-6458513
Japanese quail (*Coturnix japonica*)	XM_015870649.2	Chr:823602480-23601780
Ruddu duck (*Oxyura jamaicensis*)	XM_035333433.1	Chr8:28001757-28001057
Barn swallow (*Hirundo rustica*)	XM_040073335.2	Chr9:3604153-3603453

Table S3 lists all primers used for the plasmid construction.

Name of fragment	Length of product	Primer sequence（5'-3'）
pEGFP-JUN-promoter	2050bp	F: taccgccatgcattagttat*taat*AACCAGGGCACACACGCA<br>R: CACCATGGTGGCGACCGGTG*GATCC*ACTTATCAGCGACCGGGAGCC
pGL3-P1	745bp	F1: atttctctatcgataggtac*c*AGCCGCCGCCACCCCAGG<br>R1: ccaacagtaccggaatgcca*agctt*ACTTATCAGCGACCGGGAGCCCG
pGL3-P2	1445bp	F2: atttctctatcgataggtac*c*AGGAGGGCACAGCTGGGG<br>R1: ccaacagtaccggaatgcca*agctt*ACTTATCAGCGACCGGGAGCC
pGL3-P3	2046bp	F3: atttctctatcgataggtac*c*AACCAGGGCACACACGCA<br>R1: ccaacagtaccggaatgcca*agctt*ACTTATCAGCGACCGGGAG
Fragment 1	166bp	F1: atttctctatcgataggtac*c*AGCCGCCGCCACCCCAGG<br>D1-R: CCCTCACGTGTCGCCGCCACCGCCGCTTC
Fragment 2	589bp	D2-F: GTGGCGGCGACACGTGAGGGCTCCCCCG<br>R1:ccaacagtaccggaatgcca*agctt*ACTTATCAGCGACCGGGAGCCCG
Fragment 3	539bp	F1: atttctctatcgataggtac*c*AGCCGCCGCCACCCCAGG<br>D3-R: GGCCTTGTGGGCTGGCGGCGGCCCGGGG
Fragment 4	216bp	D4-F: CGCCGCCAGCCCACAAGGCCCCGCGCCG<br>R1:ccaacagtaccggaatgcca*agctt*ACTTATCAGCGACCGGGAGCCCG
Fragment 5	284bp	F1: atttctctatcgataggtac*c*AGCCGCCGCCACCCCAGG<br>D5-R: GTGCGGCGTGCGCGCTCCTCCGTTCGACGG
Fragment 6	468bp	D6-F: GAGGAGCGCGCACGCCGCACCTCGGCGC<br>R1:ccaacagtaccggaatgcca*agctt*ACTTATCAGCGACCGGGAGCCCG
Fragment 7	220bp	F1: atttctctatcgataggtac*c*AGCCGCCGCCACCCCAGG<br>D7-R: CCGCCTCACCTGGTACGGCCCTGAAGGTGGC
Fragment 8	526bp	D8-F: GGCCGTACCAGGTGAGGCGGCAGGCCGA<br>R1:ccaacagtaccggaatgcca*agctt*ACTTATCAGCGACCGGGAGCCCG
Fragment 9	79bp	F1: atttctctatcgataggtac*c*AGCCGCCGCCACCCCAGG<br>D9-R: GGCGCCGTGGCGCAGAGGGGCCCAGCGC

Fragment 10	673bp	D10-F: CCCCTCTGCGCCACGGCGCCGTGCGCC<br>R1:ccaacagtaccggaatgcca*agctt*ACTTATCAGCGACCGGGAGCCCG
Fragment 11	639bp	F1: atttctctatcgataggtac*c*AGCCGCCGCCACCCCAGG<br>D11-R: CCGCCGCCTCGGCCGCTGGCCCGGCCCG
Fragment 12	113bp	D12-F: GCCAGCGGCCGAGGCGGCGGTAGCCAAT<br>R1:ccaacagtaccggaatgcca*agctt*ACTTATCAGCGACCGGGAGCCCG
Fragment 13	337bp	F1: atttctctatcgataggtac*c*AGCCGCCGCCACCCCAGG<br>D13-R: CCGCGTCCCCGTGCGTTGTGTGGCGCGCAC
Fragment 14	415bp	D14-F: CACAACGCACGGGGACGCGGCGCGAGGG<br>R1:ccaacagtaccggaatgcca*agctt*ACTTATCAGCGACCGGGAGCCCG

Note: The lowercase letters are homologous arms. Italicized lowercase indicates the restriction enzyme recognition site
