# Supplementary figures and images for: A Study of JUN’s Promoter Region and Its Regulators in Chickens
Source: Genes (Basel). 2024 Oct 21;15(10):1351. doi: 10.3390/genes15101351 (PMC11508107; doi:10.3390/genes15101351)

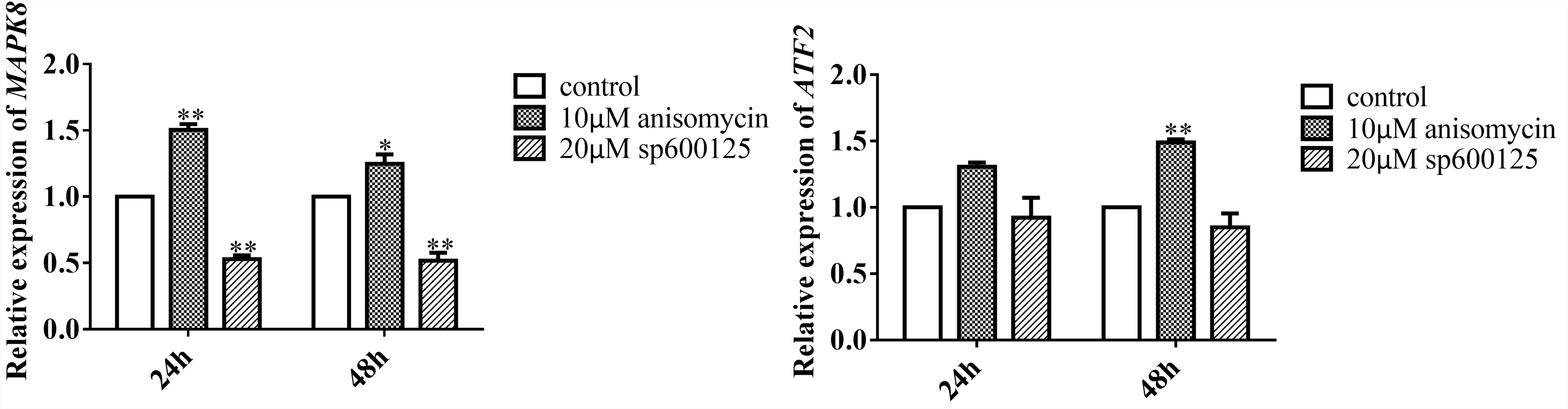

Supplement: Supplementary file 1 [file genes-15-01351-s001.zip › Supplementary Materials-Figure S1.tif]

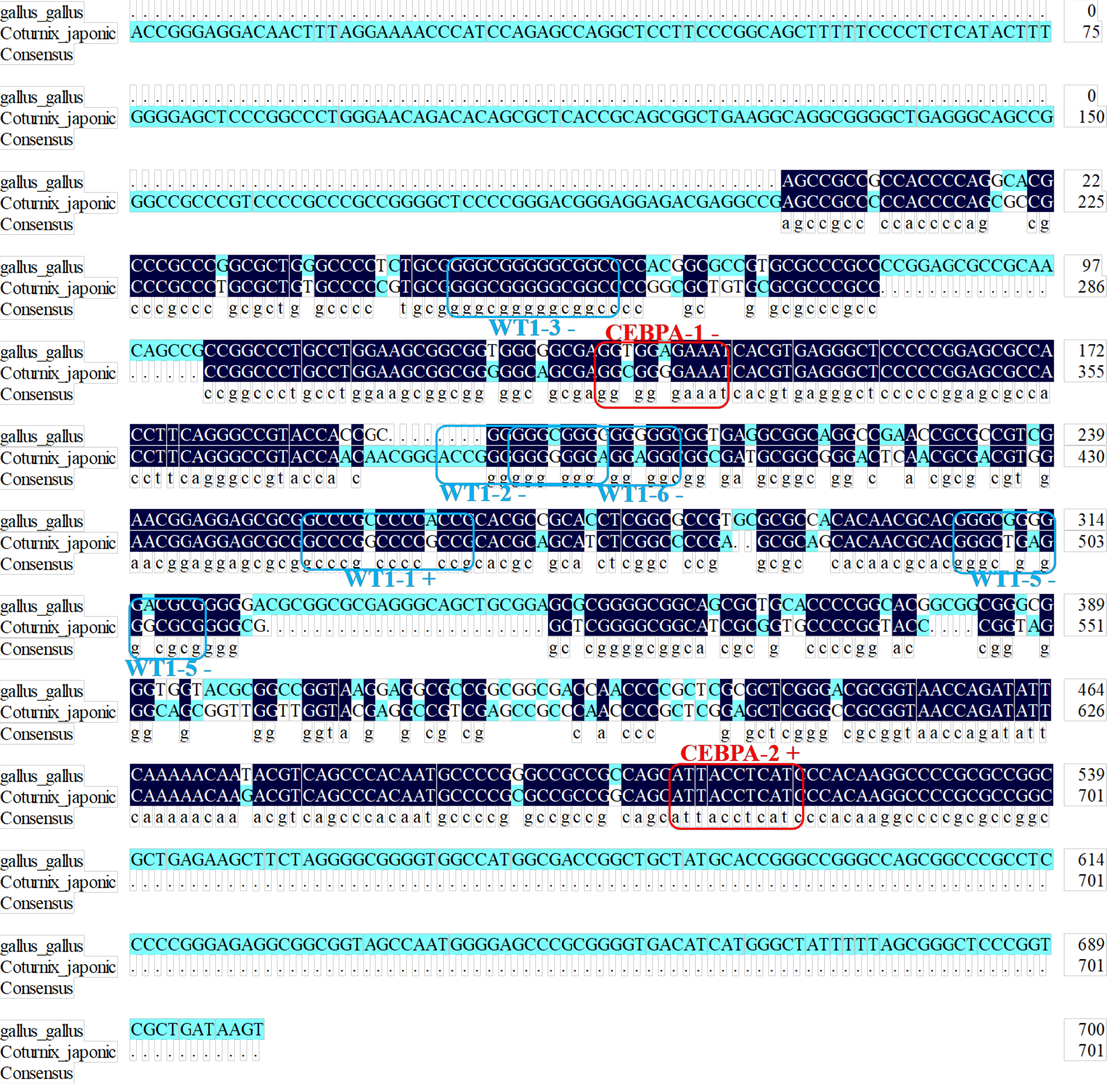

Supplement: Supplementary file 1 [file genes-15-01351-s001.zip › Supplementary Materials-Figure S2.tif]

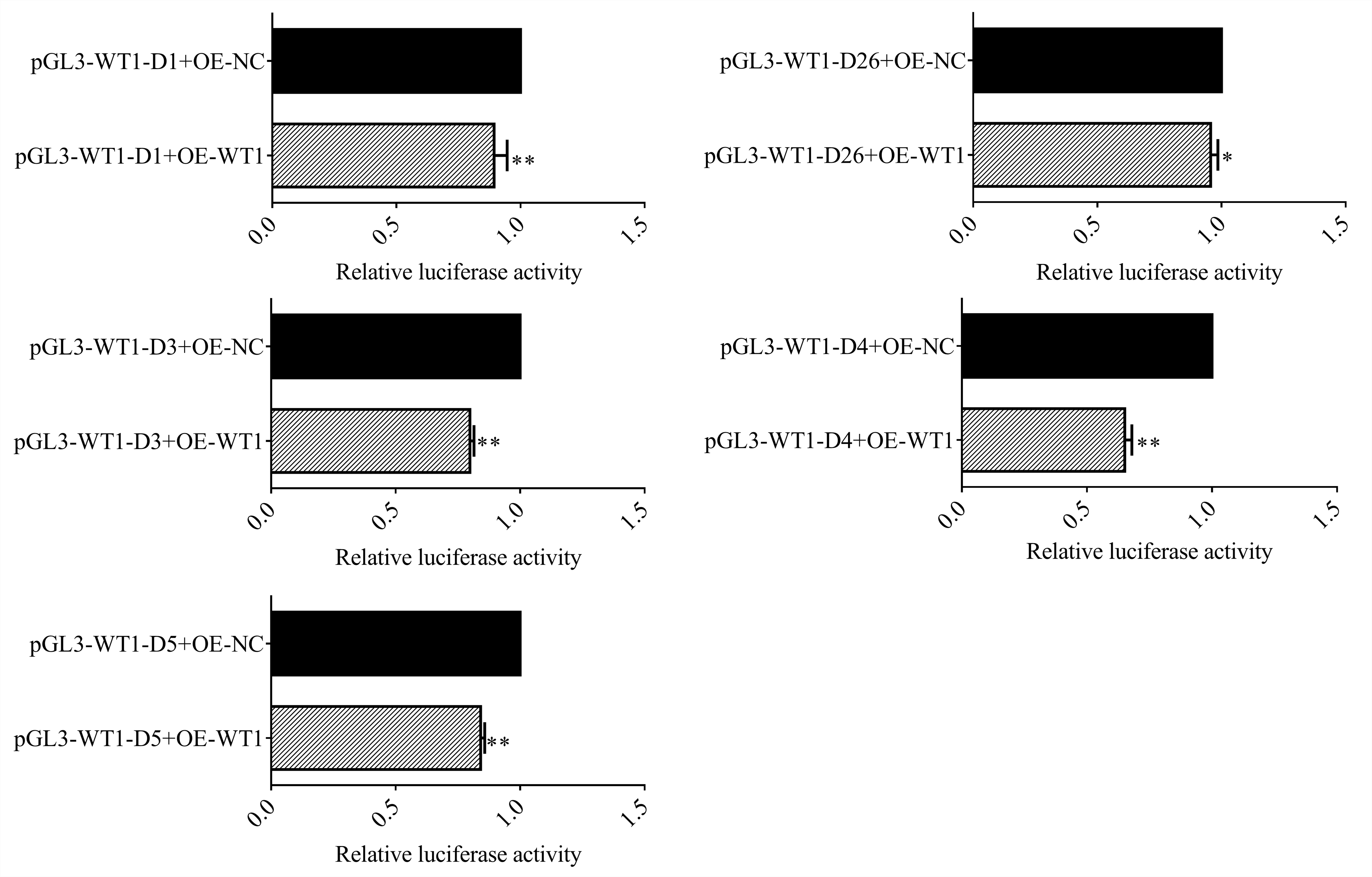

Supplement: Supplementary file 1 [file genes-15-01351-s001.zip › Supplementary Materials-Figure S4.tif]

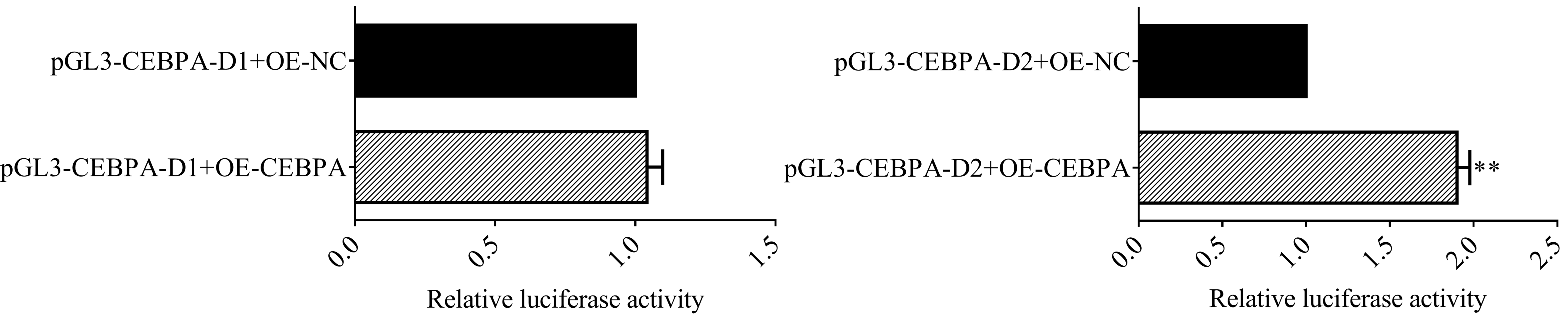

Supplement: Supplementary file 1 [file genes-15-01351-s001.zip › Supplementary Materials-Figure S5.tif]
